# Author Correction: A translational regulator MHZ9 modulates ethylene signaling in rice

**DOI:** 10.1038/s41467-023-41010-5

**Published:** 2023-08-24

**Authors:** Yi-Hua Huang, Jia-Qi Han, Biao Ma, Wu-Qiang Cao, Xin-Kai Li, Qing Xiong, He Zhao, Rui Zhao, Xun Zhang, Yang Zhou, Wei Wei, Jian-Jun Tao, Wan-Ke Zhang, Wenfeng Qian, Shou-Yi Chen, Chao Yang, Cui-Cui Yin, Jin-Song Zhang

**Affiliations:** 1 State Key Laboratory of Plant Genomics, Institute of Genetics and Developmental Biology, Innovative Academy for Seed Design, Chinese Academy of Sciences, Beijing, 100101 China; 2 College of Advanced Agricultural Sciences, University of Chinese Academy of Sciences, Beijing, 100049 China; 3 Guangdong Laboratory for Lingnan Modern Agriculture, College of Agriculture, South China Agricultural University, Guangzhou, 510642 China; 4 State Key Laboratory of Crop Gene Exploration and Utilization in Southwest China, Sichuan Agricultural University, Wenjiang, Chengdu, 611130 China; 5 MOA Key Laboratory of Pest Monitoring and Green Management, College of Plant Protection, China Agricultural University, Beijing, 100193 China

**Keywords:** Plant hormones, Plant molecular biology, Translation

Correction to: *Nature Communications* 10.1038/s41467-023-40429-0, published online 04 August 2023

The original version of this Article contained inadvertent errors in the labelling of Figure 1a. In the original version, both WT seedlings were labelled ‘Air’ and both mhz9 seedlings were labelled ‘ET’. This has been corrected so that the leftmost seedling of each genotype is labelled ‘Air’ and the rightmost seedling is labelled ‘ET’. The figure has now been corrected in the HTML and PDF versions of the article.

