# Supplementary material for: A translational regulator MHZ9 modulates ethylene signaling in rice
Source: Nat Commun. 2023 Aug 4;14:4674. doi: 10.1038/s41467-023-40429-0 (PMC10403538; doi:10.1038/s41467-023-40429-0)
Supplement: Supplementary file 1 — Supplementary Information [file 41467_2023_40429_MOESM1_ESM.pdf]

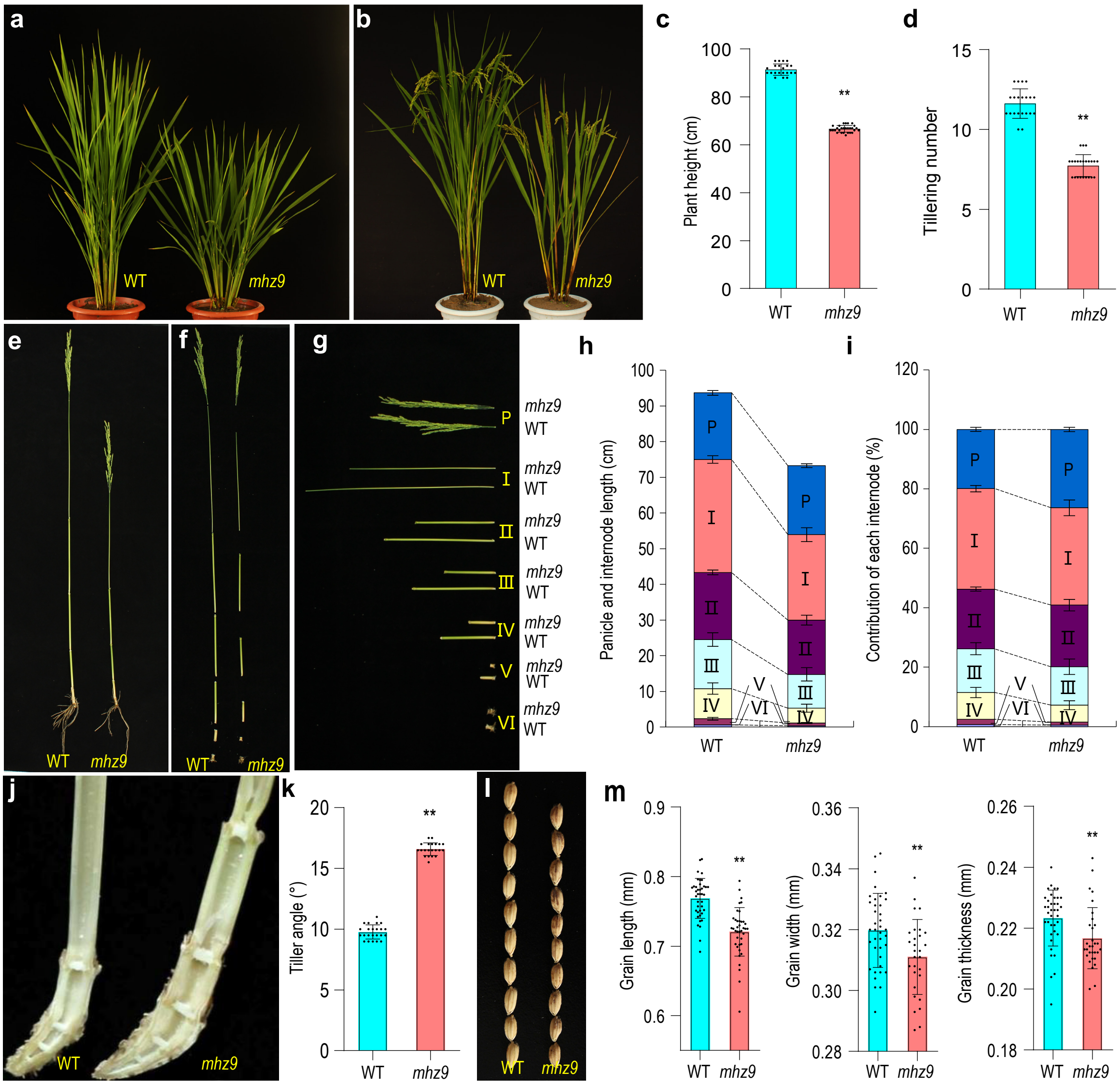

Supplementary Fig. 1 Plant phenotypes of field-grown plants of *mhz9*. Phenotypes of WT (Nipponbare) and *mhz9* at vegetative stage **(a)** and heading stage **(b)**. Plant height **(c)** and tiller number **(d)** of WT and *mhz9* plants at mature stage. Comparison of main stem **(e)**, internodes **(f)** and panicle **(g)** between WT and *mhz9*. **(h)** Lengths of panicle and internodes. **(i)** Contribution of each panicle and internode of WT and *mhz9* at percentage levels. Magnified tiller base **(j)** and maximum tiller angle **(k)** of WT and *mhz9*. **(l)** Comparison of grains between WT and *mhz9*. **(m)** Grain length (left), grain width (middle) and grain thickness (right). **(c, d, h, i, k, m)** Values are means ± SD. The asterisks indicate significant differences compared with the WT controls (**$P < 0.01$; two-tailed Student's *t*-test). Sample size ≥ 20 biologically independent samples. Source data are provided as a Source Data file.

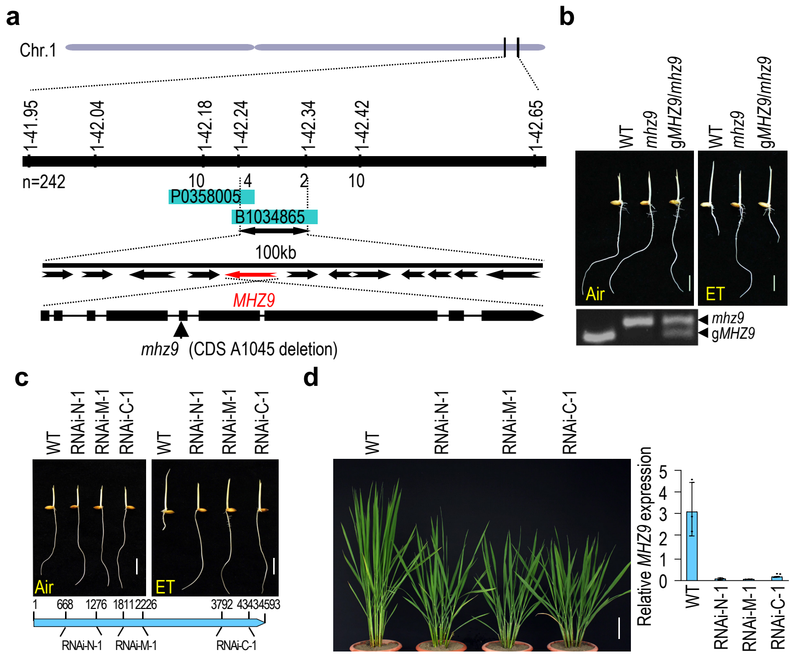

Supplementary Fig. 2 Map-based cloning, complementation analysis, RNAi analysis and comparison of the *MHZ9*-silenced plants. (a) Map-based cloning of *MHZ9*. The mutation site is indicated in schematic diagrams. **(b)** Genetic complementation. The genomic sequence of *MHZ9* was used in complementation, and the transgene is confirmed by PCR. The scale bars indicate 10 mm. **(c)** Ethylene response of transgenic seedlings with reduced *MHZ9* expression by RNAi analysis. Scale bars, 10 mm. The sequences from coding sequence (CDS) of *MHZ9* at CDS positions 668-1276 bp, 1811-2226 bp, and 3792-4343 were chosen for plasmid construction of RNAi analysis in MHZ9-RNAi-N, MHZ9-RNAi-M and MHZ9-RNAi-C, respectively. The three sites of RNAi are indicated in a schematic diagram below. **(b, c)** Seedlings were grown in the dark for 3 d in the absence (air) or presence of 10 μL/L of ethylene. **(d)** Field-grown plant phenotypes of the RNAi plants of *mhz9*. *MHZ9* expression level in these RNAi seedlings was examined by RT-qPCR. *OsActin* was used for normalization. The values are means ± SD (*n* = 3 biologically independent samples). Scale bars, 10 cm. Data are representative of three independent experiments. Source data are provided as a Source Data file.

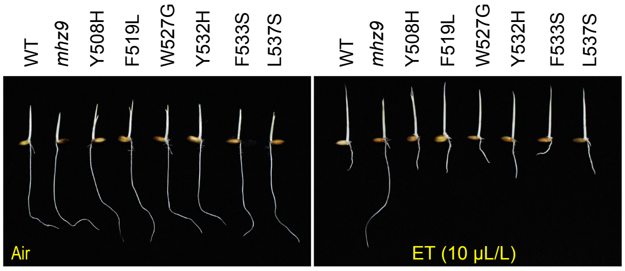

Supplementary Fig. 3 *MHZ9* gene carrying GYF-domain mutations still rescued the *mhz9* ethylene response. Ethylene response of seedlings harboring *MHZ9* genes carrying mutations of each of the six signature residues in GYF domain in *mhz9* background. Images of the representative seedlings grown in the air and in 10 μL/L of ethylene for 3 d are shown. Scale bar = 10 mm. Data are representative of three independent experiments.

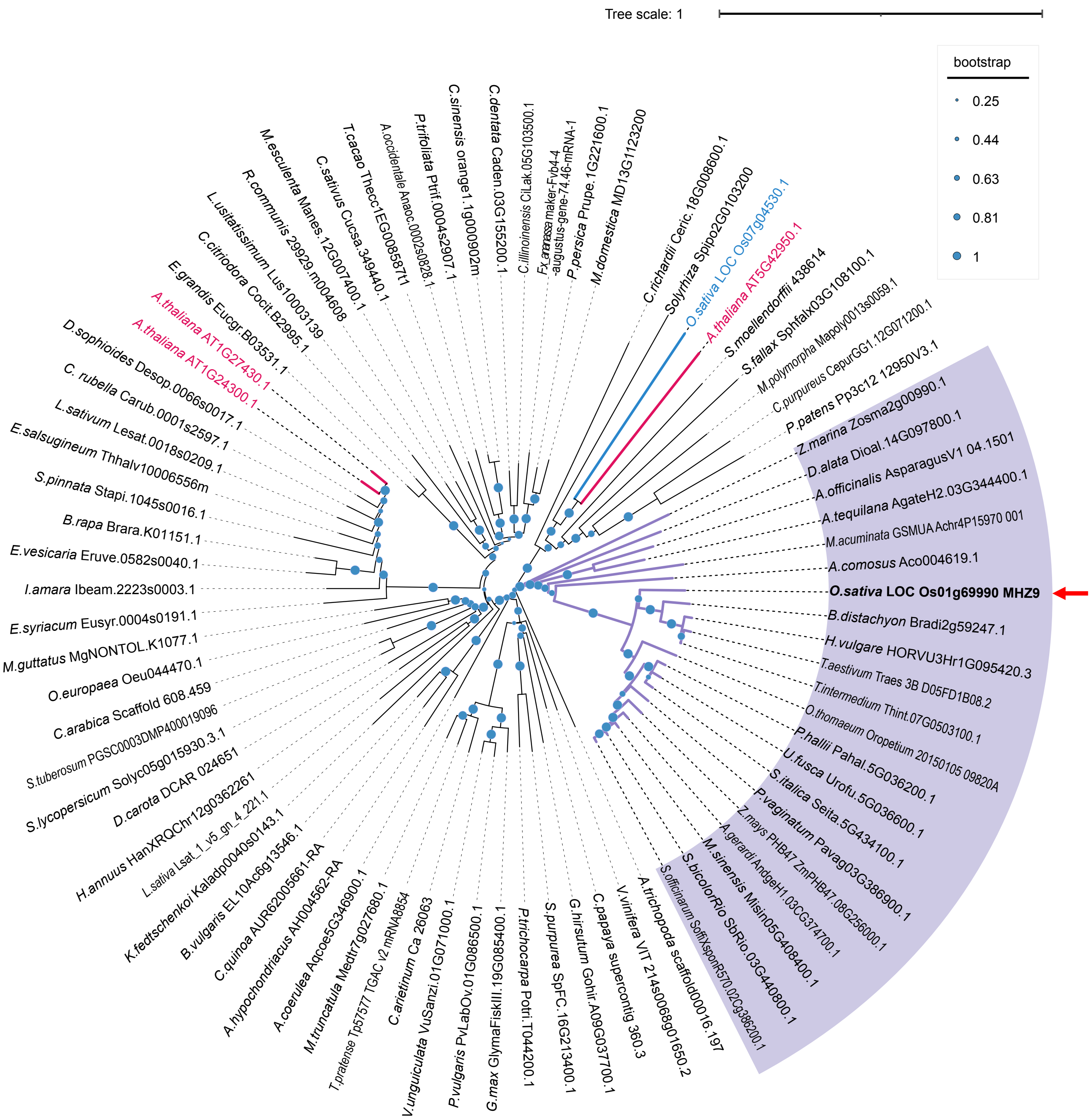

Supplementary Fig. 4 Phylogenetic analysis of MHZ9 potential homologous proteins. Sequences were aligned using MUSCLE and a neighbor-joining tree was constructed using the MEGA11.0 program with the bootstrap settings of 1000. The MHZ9 homologues from monocots and clustered with MHZ9 were indicated with a light purple background, and the MHZ9 homologues from *Arabidopsis thaliana* were indicated as red. Another MHZ9 homologue from *Oryza sativa* was indicated as blue.

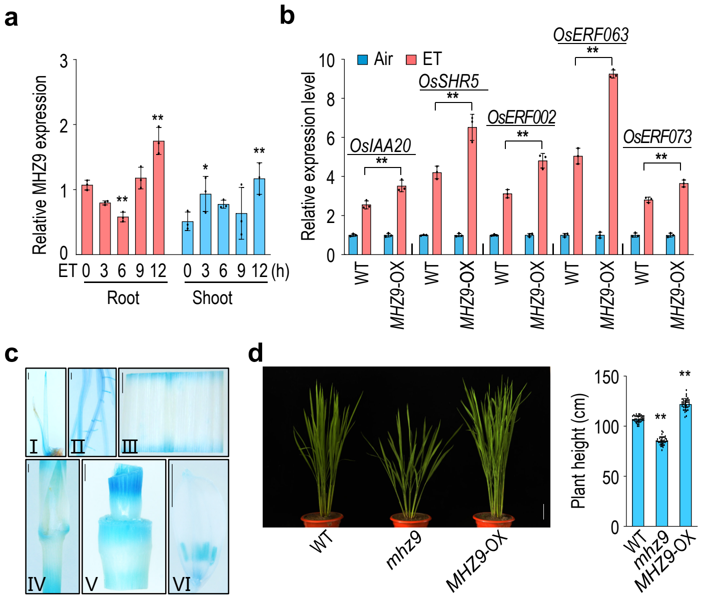

Supplementary Fig. 5 *MHZ9* expression patterns analysis and comparison of plant height in WT (Nipponbare), *mhz9*, and *MHZ9*-over-expressing plants. (a) *MHZ9* gene expression in WT roots and shoots in response to ethylene treatment. Two-day-old etiolated seedlings were treated with 10 μL/L of ethylene for different time. *OsActin* was used for normalization in RT-qPCR analysis. Values are means ± SD (n = 3 biologically independent samples). The asterisks indicate significant differences compared with the corresponding 0-h controls (*P < 0.05, **P < 0.01; two-tailed Student's t-test). **(b)** Ethylene-induced gene expression in *MHZ9*-OX roots. Total RNAs were isolated from Two-day-old etiolated seedlings treated with air or 10 μL/L of ethylene for 8 h. *OsUBQ5* was used for normalization in RT-qPCR analysis. Values are means ± SD (n = 3 biologically independent samples). The asterisks indicate significant differences compared with the corresponding WT controls (**P < 0.01; two-tailed Student's t-test). **(c)** Promoter-GUS analysis of MHZ9. At least 10 samples for each organ were observed and representative ones are presented. Ⅰ, seedling shoots; Ⅱ, seedling roots; Ⅲ, leaf blade; Ⅳ, leaf sheath; Ⅴ, node; Ⅵ, anthers and pistils of young flowers. Scale bar = 2 mm. Four-day-old seedlings were used for Ⅰ and Ⅱ, and field-grown plants in the vegetative or reproductive stages were used in Ⅲ - Ⅵ. **(d)** Height comparison of the *MHZ9*-OX plants. Images of field-grown plants are shown in the left panel, and plant heights are means ± SD (n ≥ 30 biologically independent samples). The asterisks indicate significant differences compared with the WT controls (**P < 0.01; two-tailed Student's t-test). Scale bar = 10 cm. Data are representative of three independent experiments. Source data are provided as a Source Data file.

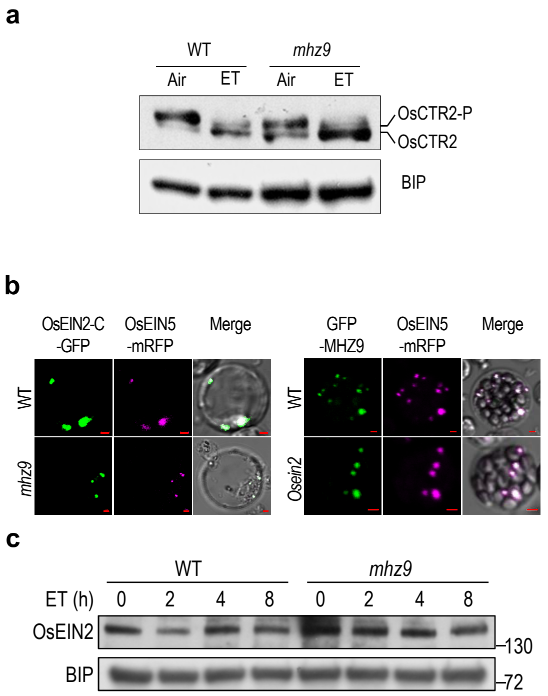

Supplementary Fig. 6 Detection of the phosphorylation status of OsCTR2 in WT and *mhz9* mutant, localization analysis and the influence of MHZ9 on accumulation of OsEIN2 protein levels. (a) Two-day-old etiolated seedlings were treated with air or 10 μL/L of ethylene for 2 h. Total proteins were isolated and immunoblotted for OsCTR2 and BIP (loading control). **(b)** Co-localization analysis of MHZ9 or OsEIN2-C with OsEIN5 in *Osein2* or *mhz9* mutant. The constructs were co-expressed in WT, *mhz9* and *Osein2* protoplasts for subcellular localization analysis. Scale bars, 2 μm. **(c)** OsEIN2 protein abundance analysis in WT and *mhz9*. Two-day-old etiolated seedlings were treated with 10 μL/L of ethylene for different time. Total proteins were isolated and immunoblotted for OsEIN2 and BIP (loading control). Data are representative of three independent experiments. Source data are provided as a Source Data file.

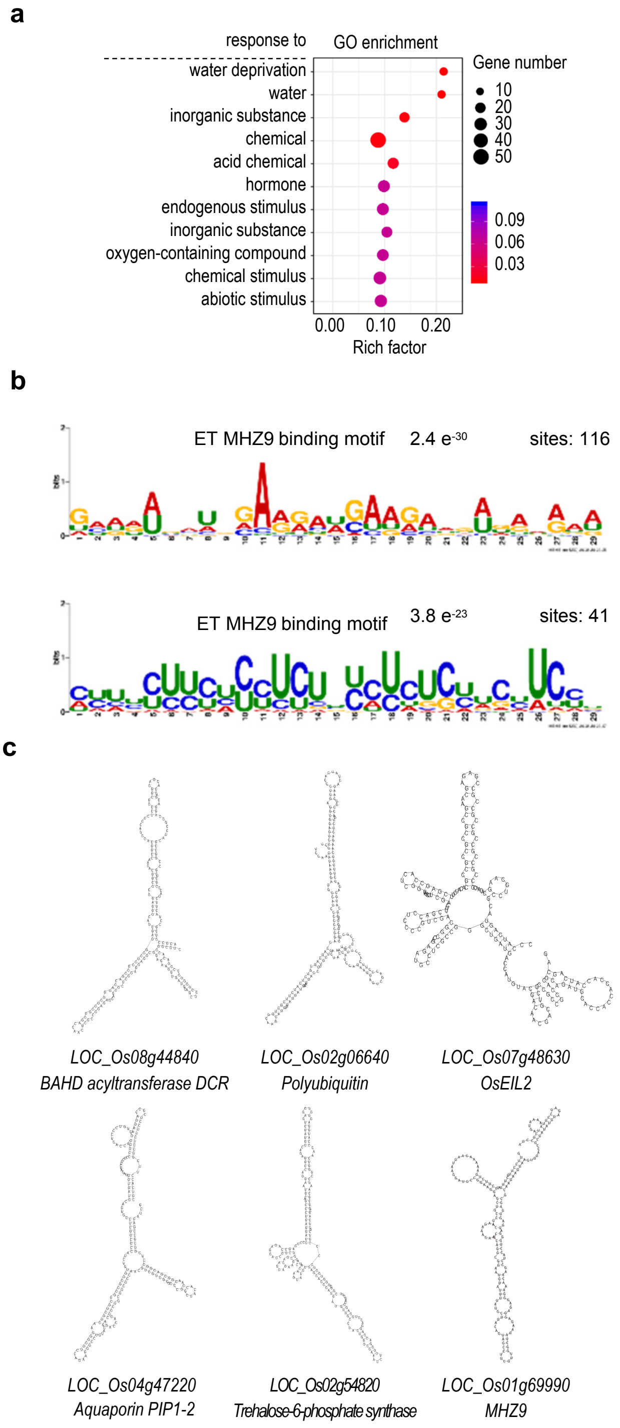

Supplementary Fig. 7 Gene Ontology (GO) analysis and putative MHZ9 binding motifs/structures of 626 genes which are overlapped in CLIP-seq and the RIP-seq analyses. (a) GO analysis of the 626 MHZ9-binding genes. **(b)** Binding motif analysis of MHZ9 in its targets. For motif search, the peak sites were extracted and uploaded to MEME website (https://meme-suite.org/meme/) for further analysis. **(c)** Predicted RNA secondary structure of MHZ9 targets. The MHZ9 binding CLIP sites with relatively high abundance, together with its 100-nt upstream and 100-nt downstream sequence, were selected for RNA secondary structure prediction using RNAfold web server. The six CLIP sites all showed stem-loop structures but without apparent similarity. Please note that the *MHZ9* mRNA (*LOC_Os01g69990*) is also bound/regulated by the MHZ9 protein itself.

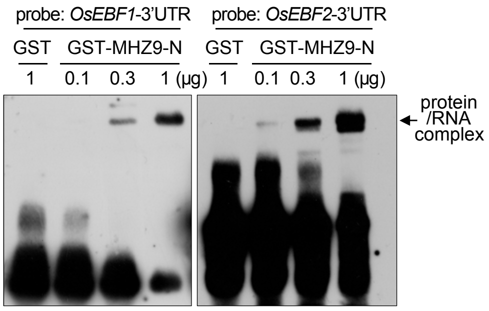

Supplementary Fig. 8 MHZ9-N exerted a dose-dependent binding activity to the 3'UTR of *OsEBF1/2* mRNA. The protein/RNA complex bands are indicated by an arrow. *OsEBF1*-3'UTR probe: CAUUACAUCAUGCUGUUUUUUUUCCAUUGCGUCGUGUUCGGAG. *OsEBF2*-3'UTR probe: CACCAUGAUUGUUUUUUAGGUUGCCGUAGUGUCCCUUGUCCUUUUUUCUUUACUGC. The experiments were repeated at least three times with similar results. And one representative set of results was shown. Source data are provided as a Source Data file.

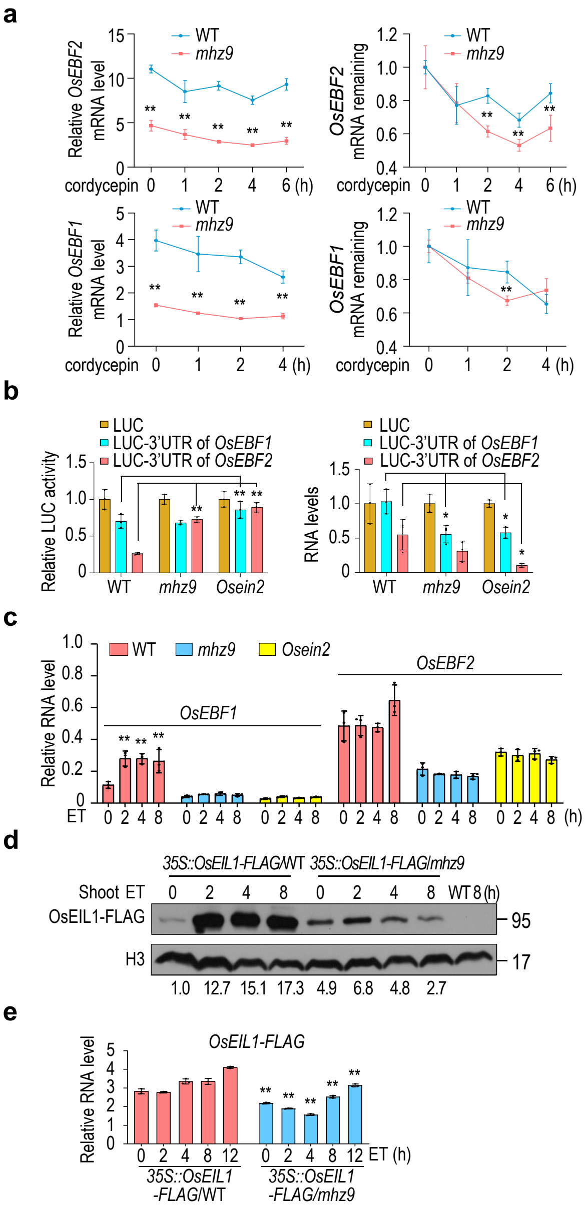

Supplementary Fig. 9 MHZ9 effects on *OsEBF1*/*2* mRNA stability, the LUC activities and RNA levels of *LUC* fusions, expression levels of *OsEBF1*/*2* and *OsEIL1*, and protein accumulation of OsEIL1 in various seedlings. (a) Quantitation of the decrease in mRNA abundance of *OsEBF1*/*2*. The WT and *mhz9* protoplasts were treated with cordycepin (100 μM) for different times. The RNA levels were detected by RT-qPCR, and *OsUBQ5* was used for normalization. The values are means ± SD (n = 4 biologically independent samples), and the asterisks indicate significant differences compared with the WT control (**P < 0.01; two-tailed Student's t-test). **(b)** MHZ9 effects on LUC activities and RNA levels of LUC fusions. The plasmid harboring both the reference *Renilla luciferase* (*R-Luc*) gene and the reporter *Firefly luciferase* gene (*F-LUC*) - 3'UTR of *OsEBF1* or *2* was transiently expressed in protoplasts. LUC activity was defined as F-LUC/R-LUC. F-LUC was used as a control (left). Relative RNA levels of *LUC* and its fused genes were analyzed through RT-qPCR. *R-LUC* was used for normalization (right). **(c)** RNA expression levels of *OsEBF1* and *OsEBF2* in WT, *mhz9* and *Osein2* etiolated seedlings. Two-day-old etiolated seedlings were treated with 10 μL/L of ethylene for different time. RNAs were extracted and the RNA levels were detected by RT-qPCR. *OsUBQ5* was used for normaliza-tion. **(d)** Accumulation of OsEIL1 protein in shoots of *35S::OsEIL1-FLAG*/WT and *35S::OsEIL1-FLAG*/mhz9 plants in response to ethylene. Etiolated two-day-old seedlings were treated with 10 uL/L ethylene for the indicated times. **(e)** RNA expression levels of *OsEIL1-FLAG* in roots of the corresponding transgenic seedlings. The RNA levels were detected with the same approach as in **(c)**. **(b, c, e)** The values are means ± SD (n = 3 biologically independent samples), and the asterisks indicate significant differences compared with the corresponding control (*P < 0.05, **P < 0.01; two-tailed Student's t-test). Data are representative of three independent experiments. Source data are provided as a Source Data file.

a

b

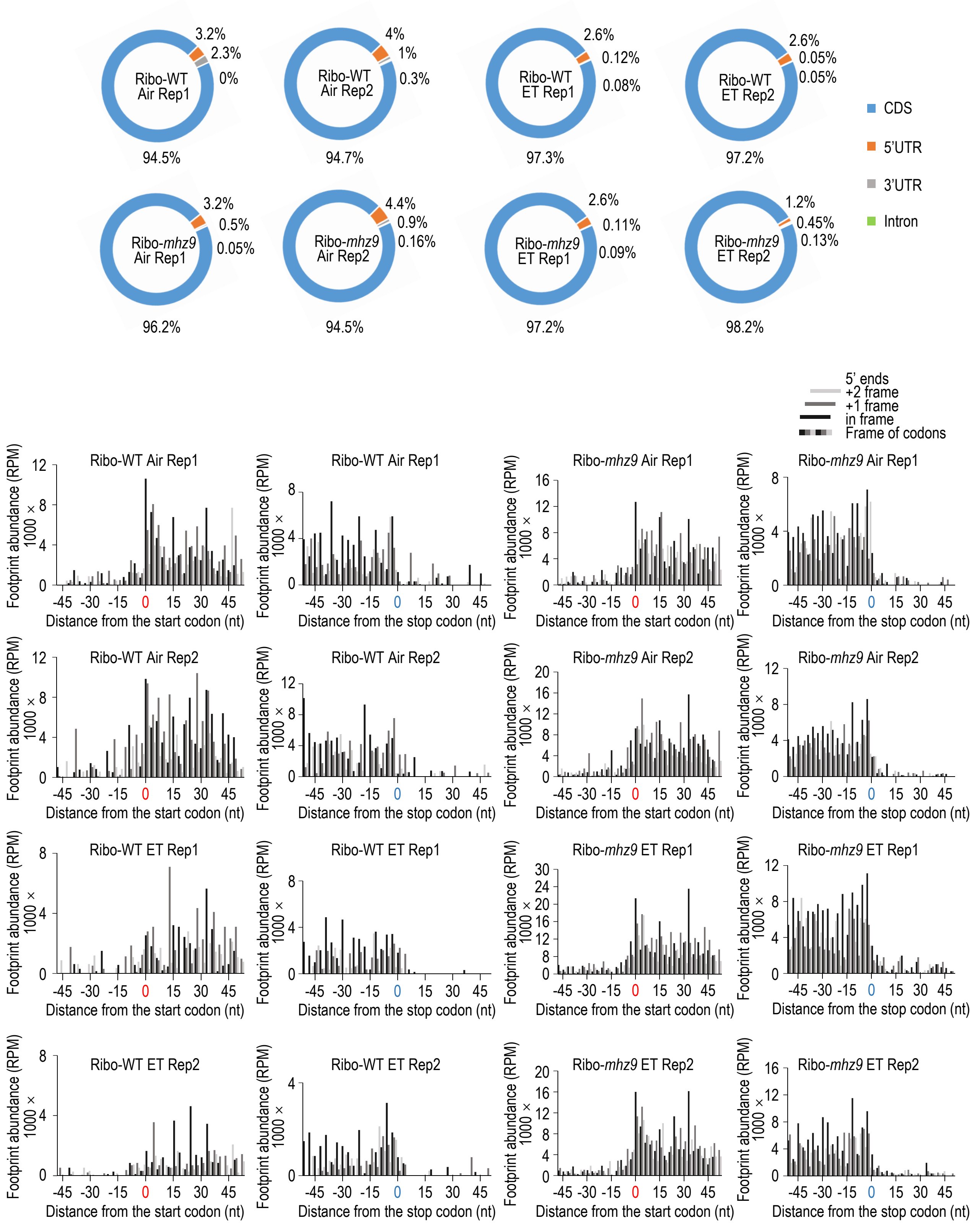

Supplementary Fig. 10 Quality assessment in Ribosome footprints analysis. (a) Distribution patterns of ribosome footprints in annotated protein and non-protein coding. **(b)** The abundance of ribosome footprints falling around the start and stop codons of protein-coding genes in the genome. The position of the 14th nucleotide in a read was used, which represents the 5'-end nucleotide in the P site of a ribosome. The frame of codons was marked with distinct colors. The start and stop codons were indicated as red and blue, respectively.
