# Supplementary material for: A translational regulator MHZ9 modulates ethylene signaling in rice
Source: Nat Commun. 2023 Aug 4;14:4674. doi: 10.1038/s41467-023-40429-0 (PMC10403538; doi:10.1038/s41467-023-40429-0)
Supplement: Supplementary file 3 — Description of Additional Supplementary Files [file 41467_2023_40429_MOESM3_ESM.docx]

**Description of Additional Supplementary Files**

**Supplementary Data 1.** **MHZ9 binding proteins identified by MHZ9-GFP IP-MS.** Results from the analysis of MHZ9 interaction protein candidates by using immunoprecipitation-mass spectrometry (IP-MS) with 2-d-old etiolated *35S::GFP-MHZ9* transgenic rice seedlings after treatment with 10 μL/L of ethylene for 8 h. The wild type (Nipponbare) rice seedlings under the same condition was used as negative control. The locus_ID and description indicate the *Oryza sativa* (RGAPv7) RGAP1- gene nomenclature and putative gene annotation. The pepcount indicates the number of matching peptides identified in MHZ9 IP-MS. Uniquepepcount indicates the number of peptide sequences unique to a protein group. Coverpercent is calculated by dividing the number of amino acids in all found peptides by the total number of amino acids in the entire protein sequence. Score for a protein is the summed score for the individual peptides. The peptides with score value no less than 20 were selected for further investgation. Expectvalue indicates the number of times expected to get a score by chance with the calculated score or better. The MS/MS data were processed with Mascot software (version 2.3.0).

**Supplementary Data 2.** **MHZ9 binding sites identified through MHZ9-N-GFP RIP-seq.** Results from the analysis of MHZ9 interaction protein candidates by using immunoprecipitation-mass spectrometry (IP-MS) with 2-d-old etiolated *35S::GFP-MHZ9* transgenic rice seedlings after treatment with 10 μL/L of ethylene for 8 h. The wild type (Nipponbare) rice seedlings under the same condition was used as negative control. The locus_ID and description indicate the Oryza sativa (RGAPv7) RGAP1- gene nomenclature and putative gene annotation. The pepcount indicates the number of matching peptides identified in MHZ9 IP-MS. Uniquepepcount indicates the number of peptide sequences unique to a protein group. Coverpercent is calculated by dividing the number of amino acids in all found peptides by the total number of amino acids in the entire protein sequence. Score for a protein is the summed score for the individual peptides. The peptides with score value no less than 20 were selected for further investgation. Expectvalue indicates the number of times expected to get a score by chance with the calculated score or better. The MS/MS data were processed with Mascot software (version 2.3.0).

**Supplementary Data 3. MHZ9 binding sites identified through MHZ9 CLIP-seq under ethylene treatment.** Results from the analysis of MHZ9 binding sites through MHZ9-CLIP- seq with *pMHZ9::MHZ9-GFP* transgenic rice plants under ethylene treatment for 8 h, and *35S::GFP* transgenic rice plant under the same treatment was used as control. Peak_ID,Chromosome, Peak_start, Peak_end, Gene_ID and Locus_ID indicate the peaks_ID (sorted by the corresponding fold-change value), chromosome, the start of MHZ9 binding peak signal, the end of MHZ9 binding peak signal, the NCBI (GCF_001433935.1) gene nomenclature and the Oryza sativa (RGAPv7) RGAP1- gene nomenclature, respectively. Aliases and Description indicate other gene symbols and putative gene annotation of the candidates. The value of MHZ9_ET or GFP_ET (control) indicate the binding signals by MHZ9-GFP or GFP under ethylene condition. The Fold_change indicates the ration of binding signals between MHZ9 and GFP control. The Single_G_padj_value indicates the false discovery rate. The reads were mapped against Oryza sativa genome from NCBI (GCF_001433935.1) with TopHat2. The bam files were sorted, indexed and calculated with SAMTools. The CLIP-seq analysis was performed with Galaxy public server (https://usegalaxy.eu/).

**Supplementary Data 4.** **MHZ9 binding genes identified through MHZ9N RIP-seq and MHZ9 CLIP-seq under ethylene treatment.** The overlapped genes (626) between MHZ9-binding genes from CLIP-seq under ethylene treatment and those from RIP-seq are regarded as stringent MHZ9 targets. Gene_ID and Locus_ID indicate the NCBI (GCF_001433935.1) gene nomenclature and the Oryza sativa (RGAPv7) RGAP1- gene nomenclature, respectively. Aliases and description indicate other gene symbols and putative gene annotation of the candidates. The RNA-seq and Ribo-seq reads rpkm indicate the transcription and translation levels of the corresponding genes, respectively.

**Supplementary Data 5.** **Summary of the reads.** Raw RNA-seq and filtered Ribo-seq reads (tRNA, rRNA depletion) were first trimmed and quality filtered with cutadapt (https://github.com/marcelm/cutadapt). Then, all these trimmed and filtered reads were mapped against Oryza sativa genome from NCBI (GCF_001433935.1) with TopHat2. For Ribo-seq and RNA-seq co-profiling, the 2-day old etiolated WT and *mhz9* seedlings were treated with or without 10 μL/L of ethylene for 4 h. Two bioreplicates of the samples were harvested for further mRNA-seq and ribosome footprints analysis.

**Supplementary Data 6.** **Correlation between biological replicates.** Raw RNA-seq and filtered Ribo-seq reads (tRNA, rRNA depletion) were first trimmed and quality filtered with cutadapt (https://github.com/marcelm/cutadapt). *Pearson* correlation coefficients between replicates were calculated to evaluate reproducibility. For Ribo-seq and RNA-seq co-profiling, the 2-day old etiolated WT and *mhz9* seedlings were treated with or without 10 μL/L of ethylene for 4 h. Two bioreplicates of the samples were harvested for further mRNA-seq and ribosome footprints analysis.

**Supplementary Data 7.** **Alterations of mRNA levels in response to ethylene in WT and *mhz9*.** The DESeq2 was used to identify differentially expressed genes at the RNA level (GCF_001433935.1_IRGSP-1.0_genomic.gff). Gene_ID, locus_ID, description, log_2_FC, P value, FDR indicate the NCBI (GCF_001433935.1) gene nomenclature, Oryza sativa (RGAPv7) RGAP1- gene nomenclature, putative gene annotations, log_2_ of fold change, P value, and false discovery rate respectively. The gene with both RNA-seq and Ribo-seq reads more than 0 RPM and with a *p*-value less than 0.05 was declared as a differentially expressed gene. The differentially expressed genes are highlighted in yellow.

**Supplementary Data 8.** **Alterations of translational levels in response to ethylene in WT and *mhz9*.** The edgeR was used to identify differentially expressed genes at the translational level (GCF_001433935.1_IRGSP-1.0_genomic.gff). Gene_ID, locus_ID, description, log_2_FC, *P* value, FDR indicate the NCBI (GCF_001433935.1) gene nomenclature, *Oryza sativa* (RGAPv7) RGAP1- gene nomenclature, putative gene annotations, log_2_ of fold change, *P* value, and false discovery rate respectively. The gene with both RNA-seq and Ribo-seq reads more than 0 RPM and with a *p*-value less than 0.05 was declared as a differentially expressed gene. The differentially expressed genes are highlighted in yellow.

**Supplementary Data 9.** **Alterations of translation efficiency levels in response to ethylene in WT and *mhz9*.** The deltaTE was used to identify differentially expressed genes at the TE level (GCF_001433935.1_IRGSP-1.0_genomic.gff). Gene_ID, locus_ID, description, log_2_FC, *P* value, FDR indicate NCBI (GCF_001433935.1) gene nomenclature, *Oryza sativa* (RGAPv7) RGAP1- gene nomenclature, putative gene annotations, the log_2_ of fold change, the *P* value, and the false discovery rate respectively. Translational efficiency (TE) was calculated by RPM (Ribo-seq)/ RPM (RNA-seq) of one gene. The gene with both RNA-seq and Ribo-seq reads more than 0 RPM and with a *p*-value less than 0.05 was declared as a differentially expressed gene. The differentially expressed genes are highlighted in yellow.

**Supplementary Data 10.** **MHZ9 effects on the mRNA, translational, and translational efficiency levels of its binding genes in response to ethylene.** Among the 626 MHZ9 binding genes, 555 genes with both RNA-seq and Ribo-seq reads > 0 RPM were selected for further analysis. The 555 MHZ9 targets (Supplementary Table 4) were compared with previously identified significantly TE-altered genes (Supplementary table 9), and totally 105 genes (in yellow background) were overlapped and these genes were regarded as MHZ9-directly regulated TE-altered genes. It should be mentioned that, based on our biochemical studies (Fig. 4-5 and Supplementary Fig. 9-10). *OsEBF1*/*2* were also proved to be MHZ9-directly regulated TE-altered genes and are included at the top of the list. Another two genes (*OsSLR1* and probable *Histidine kinase 3* ) mentioned in the discussion section are indicated in bold face. Gene_ID, locus_ID, description, Log_2_FC, *P* value, FDR indicate NCBI (GCF_001433935.1) gene nomenclature, *Oryza sativa* (RGAPv7) RGAP1- gene nomenclature, putative gene annotations, the log_2_ of fold change, the *P* value, and the false discovery rate respectively. Translational efficiency (TE) was calculated by RPM (Ribo-seq)/ RPM (RNA-seq) of one gene.

**Supplementary Data 11.** **KEGG analysis in translational, mRNA, and translation efficiency levels in response to ethylene in WT.** All the genes with pathway annotations were selected for pathway analysis. PATH, Gene_ID, locus_ID, description, logFC and *P* value indicate the pathway annotation, NCBI (GCF_001433935.1) gene nomenclature, *Oryza sativa* (RGAPv7) RGAP1- gene nomenclature, putative gene annotations, the log_2_ of fold change and the *p* value, respectively. Translational efficiency (TE) was calculated by RPM (Ribo-seq)/ RPM (RNA-seq) of one gene. The log_2_ fold changes in translational, mRNA, and TE levels of each pathway were calculated by the average of log_2_ fold changes of its contained genes, respectively. These analyses were performed in both WT and *mhz9*. The pathways with statistically significant differences compared to the average of all pathway genes were selected and shown in Figure 6i (**P*< 0.05,***P*< 0.01; Mann-Whitney U test).

**Supplementary Data 12.** **Primers used in this study.**
