# Supplementary material for: A translational regulator MHZ9 modulates ethylene signaling in rice
Source: Nat Commun. 2023 Aug 4;14:4674. doi: 10.1038/s41467-023-40429-0 (PMC10403538; doi:10.1038/s41467-023-40429-0)
Supplement: Supplementary file 5 — Reporting Summary [file 41467_2023_40429_MOESM5_ESM.pdf]

nature portfolio

Corresponding author(s): Jin-Song Zhang

Last updated by author(s): Jul 21, 2023

Reporting Summary

Nature Portfolio wishes to improve the reproducibility of the work that we publish. This form provides structure for consistency and transparency in reporting. For further information on Nature Portfolio policies, see our Editorial Policies and the Editorial Policy Checklist.

Statistics

For all statistical analyses, confirm that the following items are present in the figure legend, table legend, main text, or Methods section.

n/a	Confirmed	
☐	☒	The exact sample size (*n*) for each experimental group/condition, given as a discrete number and unit of measurement
☐	☒	A statement on whether measurements were taken from distinct samples or whether the same sample was measured repeatedly
☐	☒	The statistical test(s) used AND whether they are one- or two-sided<br>*Only common tests should be described solely by name; describe more complex techniques in the Methods section.*
☒	☐	A description of all covariates tested
☐	☒	A description of any assumptions or corrections, such as tests of normality and adjustment for multiple comparisons
☐	☒	A full description of the statistical parameters including central tendency (e.g. means) or other basic estimates (e.g. regression coefficient) AND variation (e.g. standard deviation) or associated estimates of uncertainty (e.g. confidence intervals)
☐	☒	For null hypothesis testing, the test statistic (e.g. *F*, *t*, *r*) with confidence intervals, effect sizes, degrees of freedom and *P* value noted<br>*Give P values as exact values whenever suitable.*
☒	☐	For Bayesian analysis, information on the choice of priors and Markov chain Monte Carlo settings
☒	☐	For hierarchical and complex designs, identification of the appropriate level for tests and full reporting of outcomes
☐	☒	Estimates of effect sizes (e.g. Cohen's *d*, Pearson's *r*), indicating how they were calculated

Our web collection on statistics for biologists contains articles on many of the points above.

Software and code

Policy information about availability of computer code

Data collection	Zeiss Axio Imager Z2 was used to capture fluorescent signals.<br>The polysome profiling signals were detected using a piston gradient fractionator (Biocomp, B152-002) at 254-nm UV absorbance.
Data analysis	Statistical analysis of the root length and coleoptile length in ethylene responses were performed by using EXCEL 2010.<br>For RNA-seq, RIP-seq, CLIP-seq and Ribo-seq data analysis: Bowtie 2 v2.4.2, cutadapt v2.10, TopHat v2.0.10, Samtools v1.8, PEAKachu v0.1.0, BEDtools v2.29.2, DEseq2 v 2.11.40.7, edgeR v3.36, DTEG.R.<br>RibORF software was used to identify ORFs and perform quality assessment of Ribo-seq data.<br>For MHZ9 binding motif search: Samtools v1.8, MEME website (https://meme-suite.org/meme/).<br>For MHZ9 KEGG analysis: TBtools v1.098667.

For manuscripts utilizing custom algorithms or software that are central to the research but not yet described in published literature, software must be made available to editors and reviewers. We strongly encourage code deposition in a community repository (e.g. GitHub). See the Nature Portfolio guidelines for submitting code & software for further information.

Data

Policy information about availability of data

All manuscripts must include a data availability statement. This statement should provide the following information, where applicable:

- Accession codes, unique identifiers, or web links for publicly available datasets
- A description of any restrictions on data availability
- For clinical datasets or third party data, please ensure that the statement adheres to our policy

The RNA-IP Seq data generated in this study have been deposited in the NCBI/Sequence Read Archive (SRA) database under accession code PRJNA890558 [https://dataview.ncbi.nlm.nih.gov/object/PRJNA890558]. The CLIP-Seq data generated in this study have been deposited in the NCBI/Sequence Read Archive (SRA) database under accession code PRJNA896502 [https://dataview.ncbi.nlm.nih.gov/object/PRJNA896502]. The Ribosome footprints Seq data generated in this study have been deposited in the NCBI/Sequence Read Archive (SRA) database under accession code PRJNA891450 [https://dataview.ncbi.nlm.nih.gov/object/PRJNA891450]. The mass spectrometry proteomics data generated in this study have been deposited in the ProteomeXchange Consortium via the PRIDE partner repository under accession code PXD041240 [https://www.ebi.ac.uk/pride/archive/projects/PXD041240/]. Source data are provided with this paper. All other study data are included in the article and/or supporting information.

Research involving human participants, their data, or biological material

Policy information about studies with human participants or human data. See also policy information about sex, gender (identity/presentation), and sexual orientation and race, ethnicity and racism.

Reporting on sex and gender	N/A.
Reporting on race, ethnicity, or other socially relevant groupings	N/A.
Population characteristics	N/A.
Recruitment	N/A.
Ethics oversight	N/A.

Note that full information on the approval of the study protocol must also be provided in the manuscript.

Field-specific reporting

Please select the one below that is the best fit for your research. If you are not sure, read the appropriate sections before making your selection.

[x] Life sciences [ ] Behavioural & social sciences [ ] Ecological, evolutionary & environmental sciences

For a reference copy of the document with all sections, see nature.com/documents/nr-reporting-summary-flat.pdf

Life sciences study design

All studies must disclose on these points even when the disclosure is negative.

Sample size	No sample size calculation was performed. Sample size of all experiments was decided based on the feasibility of sample collection. The results are reliable and reproducible.
Data exclusions	No data were excluded from our analyses.
Replication	The number of replication are indicated in the figure legends.
Randomization	Samples were arranged randomly in related experiments.
Blinding	The blinding was not applied. Because all the experiments were performed without prior knowledge of the final outcome.

Reporting for specific materials, systems and methods

We require information from authors about some types of materials, experimental systems and methods used in many studies. Here, indicate whether each material, system or method listed is relevant to your study. If you are not sure if a list item applies to your research, read the appropriate section before selecting a response.

Materials & experimental systems

n/a	Involved in the study	
☐	☒	Antibodies
☒	☐	Eukaryotic cell lines
☒	☐	Palaeontology and archaeology
☒	☐	Animals and other organisms
☒	☐	Clinical data
☒	☐	Dual use research of concern
☐	☒	Plants

Methods

n/a	Involved in the study	
☒	☐	ChIP-seq
☒	☐	Flow cytometry
☒	☐	MRI-based neuroimaging

Antibodies

Antibodies used	In co-immunoprecipitation and Western-blot analyses, antibodies against OsEIN2 (1:10,000) and OsCTR2 (1:10,000) were prepared in our laboratory.<br>And anti-GFP (7G9) (1:5,000; M2004), anti-Actin (1:5,000; cytoplasm marker; 26F7), anti-Myc (HRP Conjugated, 1:5,000; M20019), anti-FLAG (3B9) (1:5,000; M20008) and secondary goat anti-rabbit or anti-mouse-IgG-horseradish peroxidase (M210011, M210021) antibodies were purchased from Abmart. Anti-histone H3 (1:10,000; nuclear marker; AS10 710) antibody was purchased from Agrisera.
Validation	The specificity of antibody in rice against OsEIN2 was verified in previous publication: Ma, B. et al. (2018). PNAS. 115, 2520-2525.<br>The specificity of antibody in rice against OsCTR2 was verified in previous publication: Zhao, H. et al. (2020). Plant Cell, 32:1626-1643.<br>Validation statement for anti-GFP, anti-Actin, acti-Myc, anti-FLAG and secondary goat anti-rabbit or anti-mouse-IgG-horseradish peroxidase antibodies can be found at the product website http://www.ab-mart.com.cn/.

Dual use research of concern

Policy information about dual use research of concern

Hazards

Could the accidental, deliberate or reckless misuse of agents or technologies generated in the work, or the application of information presented in the manuscript, pose a threat to:

No	Yes	
☒	☐	Public health
☒	☐	National security
☒	☐	Crops and/or livestock
☒	☐	Ecosystems
☒	☐	Any other significant area

Experiments of concern

Does the work involve any of these experiments of concern:

No	Yes	
☒	☐	Demonstrate how to render a vaccine ineffective
☒	☐	Confer resistance to therapeutically useful antibiotics or antiviral agents
☒	☐	Enhance the virulence of a pathogen or render a nonpathogen virulent
☒	☐	Increase transmissibility of a pathogen
☒	☐	Alter the host range of a pathogen
☒	☐	Enable evasion of diagnostic/detection modalities
☒	☐	Enable the weaponization of a biological agent or toxin
☒	☐	Any other potentially harmful combination of experiments and agents
